# “Prion-like” seeding and propagation of oligomeric protein assemblies in neurodegenerative disorders

**DOI:** 10.3389/fnins.2024.1436262

**Published:** 2024-08-05

**Authors:** Silvia Zampar, Sonja E. Di Gregorio, Gustavo Grimmer, Joel C. Watts, Martin Ingelsson

**Affiliations:** ^1^Krembil Brain Institute, University Health Network, Toronto, ON, Canada; ^2^Tanz Centre for Research in Neurodegenerative Diseases, University of Toronto, Toronto, ON, Canada; ^3^Department of Biochemistry, University of Toronto, Toronto, ON, Canada; ^4^Department of Laboratory Medicine and Pathobiology, University of Toronto, Toronto, ON, Canada; ^5^Department of Medicine, University of Toronto, Toronto, ON, Canada; ^6^Department of Public Health/Geriatrics, Rudbeck Laboratory, Uppsala University, Uppsala, Sweden

**Keywords:** neurodegeneration, oligomers, seeding, propagation, misfolding

## Abstract

Intra- or extracellular aggregates of proteins are central pathogenic features in most neurodegenerative disorders. The accumulation of such proteins in diseased brains is believed to be the end-stage of a stepwise aggregation of misfolded monomers to insoluble cross-β fibrils via a series of differently sized soluble oligomers/protofibrils. Several studies have shown how α-synuclein, amyloid-β, tau and other amyloidogenic proteins can act as nucleating particles and thereby share properties with misfolded forms, or strains, of the prion protein. Although the roles of different protein assemblies in the respective aggregation cascades remain unclear, oligomers/protofibrils are considered key pathogenic species. Numerous observations have demonstrated their neurotoxic effects and a growing number of studies have indicated that they also possess seeding properties, enabling their propagation within cellular networks in the nervous system. The seeding behavior of oligomers differs between the proteins and is also affected by various factors, such as size, shape and epitope presentation. Here, we are providing an overview of the current state of knowledge with respect to the “prion-like” behavior of soluble oligomers for several of the amyloidogenic proteins involved in neurodegenerative diseases. In addition to providing new insight into pathogenic mechanisms, research in this field is leading to novel diagnostic and therapeutic opportunities for neurodegenerative diseases.

## 1 Introduction

A wealth of studies in the last three decades strongly suggest that most neurodegenerative disorders are driven by mismetabolism and accumulation of specific misfolded proteins in the brain. In Parkinson’s disease (PD), dementia with Lewy bodies (DLB) and multiple system atrophy (MSA) aggregates of α-synuclein (α-syn) are the main components of intracellular Lewy bodies and Lewy neurites. These disorders are thus referred to as α-synucleinopathies (Henderson et al., 2019). In Alzheimer’s disease (AD), aggregated amyloid-β (Aβ) forms extracellular plaques, and the tau protein accumulates as intracellular insoluble filaments known as neurofibrillary tangles (NFTs). For this reason, AD can be described as both an amyloidopathy and a tauopathy (Hardy and Higgins, 1992). Tau pathology is also displayed in progressive supranuclear palsy (PSP), Pick’s disease, corticobasal degeneration (CBD), frontotemporal dementia (FTD) and certain subgroups of frontotemporal lobar degeneration (FTLD) (Kovacs, 2017). Other groups of FTLD, alongside most cases of amyotrophic lateral sclerosis (ALS) instead show accumulation of TAR DNA binding protein-43 (TDP-43) (Liao et al., 2022). Ubiquitinated and hyperphosphorylated carboxyl (C)-terminal fragments of TDP-43 generate cellular inclusions in both neuronal and glial cells (Mackenzie and Rademakers, 2008), while the aggregation of toxic superoxide dismutase 1 (SOD1) has been linked to some variants of ALS (Abati et al., 2020). Finally, in Huntington’s disease (HD), the trinucleotide CAG expansion in the huntingtin gene (*HTT*) lead to the production and intracellular accumulation of abnormal huntingtin (Htt) protein with N-terminal polyglutamine repeat expansion inside neurons (Walker, 2007).

Similar to *HTT*, disease-causing mutations in the genes encoding for several of the other aggregating proteins have also been identified. Thus, abnormal levels or variants of these proteins are likely to be central to the respective disorders. Several mutations in *SNCA* (encoding for α-syn) (reviewed in Nussbaum, 2018), *APP* and *PSEN1/PSEN2* (encoding for the amyloid precursor protein (APP) and the presenilins that are involved in APP processing) (reviewed in Ayodele et al., 2021), *MAPT* (encoding for tau) (Ghetti et al., 2015) and *TARDBP* (encoding for TDP43) (Mackenzie and Rademakers, 2008) cause hereditary forms of PD/DLB, AD, FTD and ALS, respectively. Such pathogenic mutations generally lead to increased aggregate formation due to higher levels and/or to enhanced aggregation propensities of the mutant protein. Most of the above-mentioned neurodegenerative proteins can self-assemble and form deposits that display amyloidogenic properties and stain positive for Congo red and/or its derivatives (Westermark et al., 1999). During the aggregation process, differently sized and soluble multimers and prefibrils of the amyloidogenic protein are formed. In this context, studies have indicated that soluble prefibrillar oligomers, rather than insoluble fibrils, are particularly pathogenic (Caughey and Lansbury, 2003; Lesne and Kotilinek, 2005; Gadad et al., 2011).

Misfolding of the native protein into a pathogenic conformation is a key feature of the aggregation process in all neurodegenerative diseases (Cerda-Costa et al., 2007; Soto and Pritzkow, 2018). A very well studied example comes from a set of specific transmissible human neurodegenerative disorders, known as prion diseases, where the pathological protein presents unique mechanistic characteristics. In disorders such as Creutzfeldt-Jakob disease (CJD), Kuru, Gerstmann-Sträussler-Scheinker disease and familial fatal insomnia, also classified as transmissible spongiform encephalopathies (TSEs), the soluble prion protein (PrP^C^) acquires an abnormal conformation, known as PrP^Sc^, which is protease resistant and acts as a seed for the misfolding of physiological PrP^C^ (Beck et al., 1969; Gibbs and Gajdusek, 1969; Prusiner, 1982). The peculiarity of prion diseases is their transmissibility, which depends on seeding and propagation of the infectious PrP^Sc^ protein aggregates. The prion aggregates self-replicate and propagate in the affected brain, spreading the misfolding and aggregation process until a toxicity threshold for clinical manifestation is reached (Prusiner et al., 1998; Soto, 2012).

Comparable to the classical prion diseases, similar mechanisms could also be central pathogenic features in other neurodegenerative disorders, given the seeding ability of misfolded protein aggregates. Several *in vitro* and *in vivo* studies have shown how α-syn, Aβ and tau can be transmitted via a “prion-like” mechanism in cellular and animal models (Soto, 2012; Goedert, 2015; Stopschinski and Diamond, 2017). While there is robust evidence that many amyloidogenic proteins can act as nucleating particles and spread within cellular networks, it remains unclear how the different species (e.g., monomers, oligomers and fibrils) in the respective aggregation cascades contribute to the seeding and propagation of such proteins in neurodegenerative diseases. Given their key role in neurodegeneration, oligomers may be central in these processes. In this review, we will highlight the importance of oligomers in the pathogenesis of several neurodegenerative disorders and provide examples on how such intermediately sized species contribute to seeding and propagation of pathology in various central nervous system (CNS) proteinopathies.

## 2 Amyloidogenic proteins aggregate via the formation of misfolded oligomers/protofibrils

**Figure 1:**
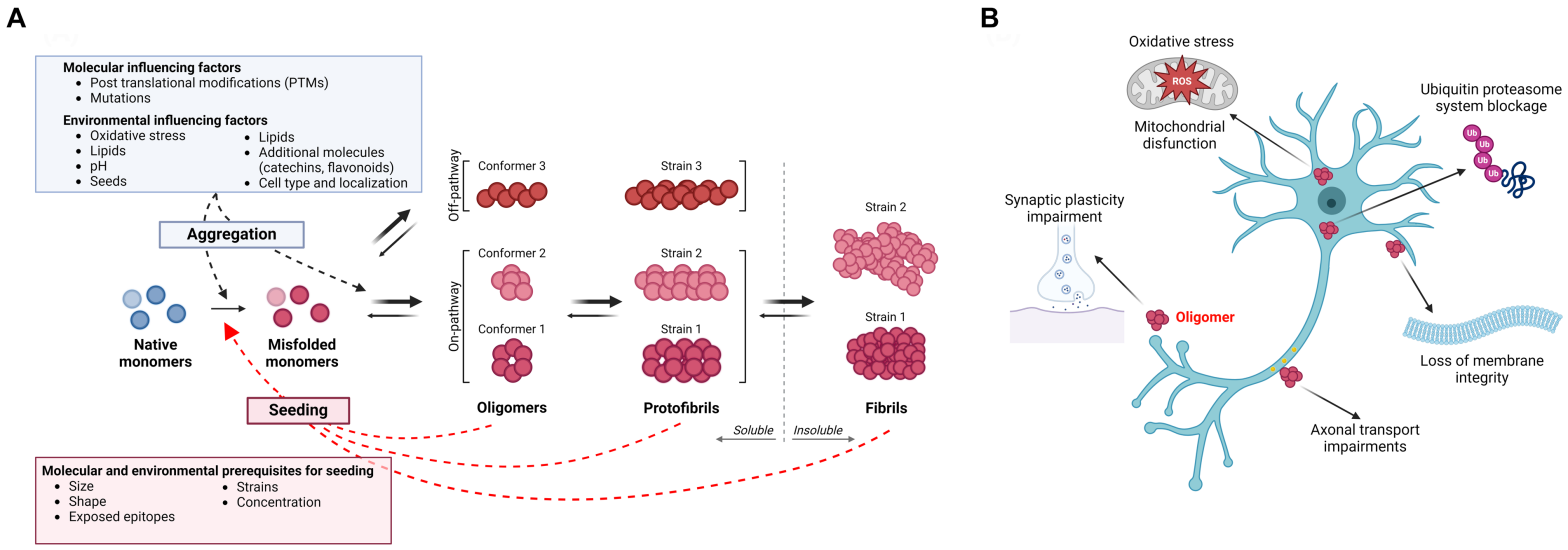
**(A)** Amyloidogenic protein aggregation process and influencing factors. The aggregation process of amyloidogenic proteins follows specific kinetics and the different species are usually present in an equilibrium. In disease, a native monomer assumes a misfolded pathological conformation and aggregates into soluble higher molecular weight species, such as oligomers and protofibrils, which are believed to be the main toxic species in several neurodegenerative diseases. These processes appear to be influenced by several molecular and environmental factors. Oligomers/protofibrils can assume different conformations, leading to the generation of different strains of the same protein. Further aggregation of oligomers and protofibrils leads to the formation of insoluble amyloid fibrils. Next to such on-pathway oligomers, accumulating evidence supports the existence of off-pathway oligomers, which do not aggregate further into insoluble fibrils. Both soluble species and insoluble fibrils of different amyloidogenic proteins might possess “prion-like” nucleating properties, templating the misfolding of a native monomer into a pathogenic conformation. As for the aggregation process, seeding is influenced by molecular properties of the nucleating particles as well as by environmental factors. **(B)** Toxicity of soluble oligomers. Oligomers have been found to exert their toxicity targeting several processes. The most common and shared effects are mitochondrial dysfunction and generation of reactive oxygen species (ROS), impairment of synaptic plasticity and axonal transport, disruption of membrane integrity and blockage of the ubiquitin proteasome system. Created with BioRender.com.

**Figure 2:**
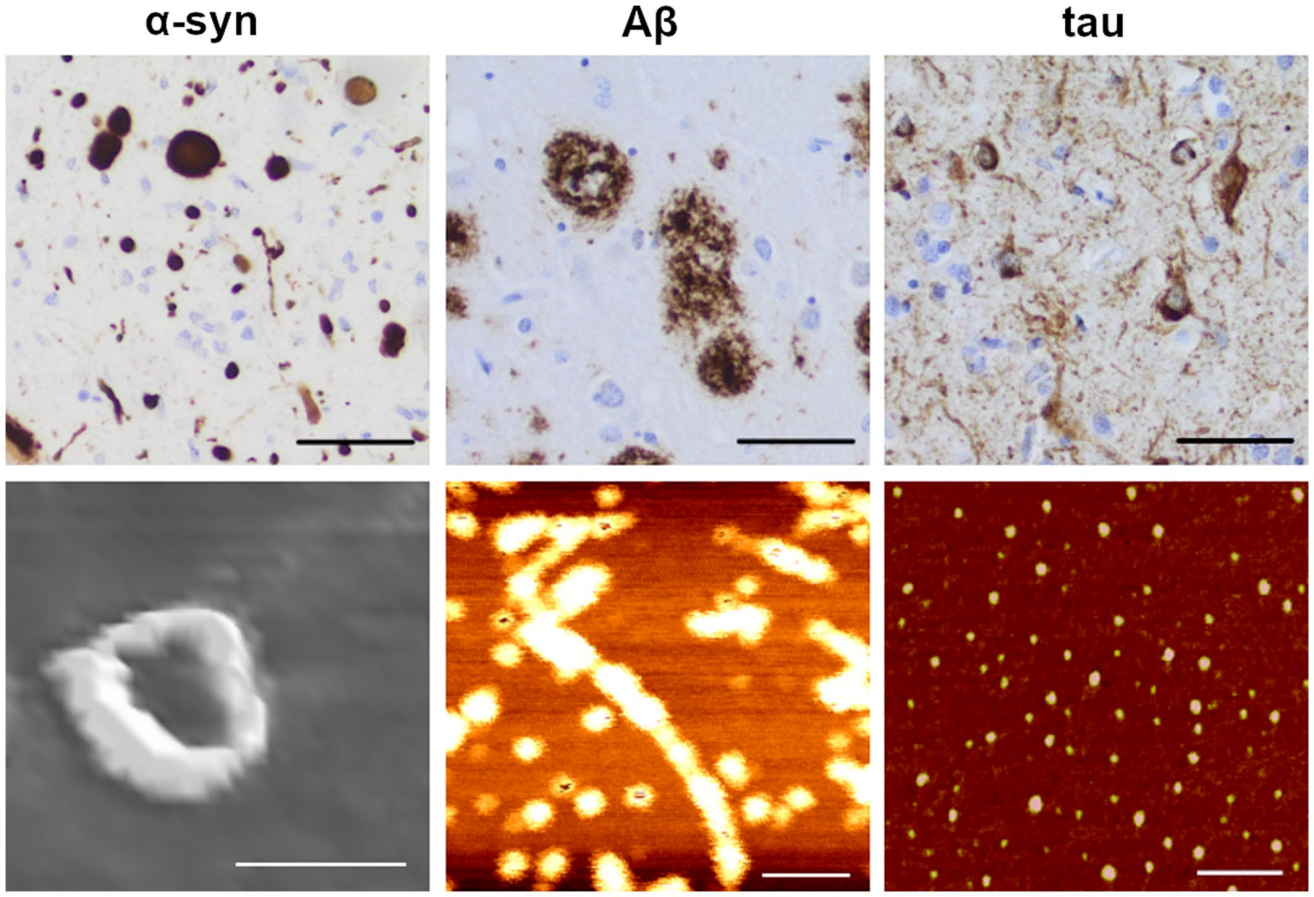
Immunohistochemical staining and atomic force microscopy images of amyloidogenic proteins. Upper row: example images of α-synuclein (α-syn; left), amyloid-β (Aβ; middle) and hyperphosphorylated tau (right) pathology in an AD brain (scale bars: 50 μm), visualized by immunohistochemical staining with specific antibodies as previously described (Libard et al., 2022; anti α-syn: KM51, Novocastra; anti Aβ: 4G8, Biolegend; anti phospho-tau: AT8, Fisher Scientific-Invitrogen). Lower row: Atomic force microscopy (AFM) images of oligomeric forms of the corresponding proteins. Protofibrils of α-synuclein were induced with 4-hydroxy-trans-2-nonenal (HNE; scale bar: 50 nm) from recombinant E46K monomers. Protofibrils of Aβ were induced from synthetic 1–42 wt peptide (scale bar: 100 nm). Oligomers of tau were induced from wt 2N4R tau (scale bar: 100 nm). The immunohistochemical images were kindly provided by Dr. Sylwia Libard. The AFM images were kindly provided by Dr. Mikael Karlsson (α-synuclein and Aβ) and the Kayez lab (tau).

The formation of insoluble aggregates proceeds via a stepwise process in which the respective proteins transform from physiological to misfolded monomers and then sequentially polymerize into dimers/smaller oligomers to larger oligomers/protofibrils before they eventually deposit as *bona fide* fibrils (Mastrangelo et al., 2006; Finder and Glockshuber, 2007; Figure 1A). The insoluble aggregates consist of highly ordered fibrils of misfolded proteins, with individual polypeptide chains arranged in an orientation perpendicular to the axis of the fiber, a structure known as a cross-β sheet (Serpell et al., 2000; Fitzpatrick et al., 2013). Many proteins can fold into multiple β-sheet-rich amyloid conformations, each with its distinct characteristics (reviewed in Koo et al., 1999) (examples of immunohistochemically stained protein aggregates are shown in Figure 2, upper panel).

The aggregation of amyloidogenic proteins and the formation of insoluble fibrils are a thermodynamically favorable process, where often intrinsically disordered and unstable monomers fold into a β-sheet conformation that provides a lower energetic state. The various aggregation steps from monomers to oligomers and protofibrils are generally believed to be reversible and follow measurable aggregation kinetics (Meisl et al., 2018). A widely accepted kinetic model to describe the misfolding and aggregation process is the seeding-nucleation model, composed of a slow nucleation phase followed by a subsequent rapid elongation phase (Jarrett and Lansbury, 1993; Meisl et al., 2017). In the nucleation phase, which is the rate-limiting step, a stable seed (or nucleus) of polymerized protein is formed. Once seeds are present, the monomeric protein can be incorporated and contribute to a rapid growth of the polymer (Jarrett and Lansbury, 1993; Soto et al., 2006). As insoluble amyloid deposits are thermodynamically stable, they could be considered irreversible, although release of soluble species from such insoluble aggregates has been proposed to occur (Bigi et al., 2022).

Oligomers, the highly dynamic soluble intermediates of the various aggregation cascades, seem to be of pathogenic relevance. There is still not a consensus of what can be considered an oligomer, as this term has been used to identify a broad range of differently sized or soluble species and can differ from one amyloidogenic protein to another. For the purpose of this review, we define oligomers as protein assemblies consisting of at least two monomeric units, representing a soluble intermediary between monomers and insoluble fibrils. Thus, early formed species in the aggregation process would be dimers, trimers and tetramers, whereas later-formed oligomers would consist of a larger number of protein monomers. While some studies have further classified oligomers into low molecular weight (2–5 monomer units) versus high molecular weight (6–600 monomer units) protein assemblies (Walsh and Selkoe, 2007; Ono et al., 2014; Iljina et al., 2016; Siddiqi et al., 2019), the ranges are likely to differ between proteins and studies. Oligomers assembled during the nucleation phase of the aggregation process, and which can be incorporated into cross β-sheet fibrils, are known as on-pathway oligomers, while those that are formed as terminal products outside the fibrillation process are referred to as off-pathway oligomers (Breydo and Uversky, 2015). Further addition of monomeric units can lead to the formation of protofibrils, bead-like structures measuring up to 200 nm in length (Walsh et al., 1997; Wong et al., 1997). For Aβ, the formation of protofibrils generally occurs during the lag phase and these assemblies tend to dissociate when incubated in buffers, which suggests a structural instability compared to amyloid fibrils (Harper et al., 1997). Because of the unstable and often transient nature of oligomers and protofibrils, the isolation and characterization of such species are particularly challenging. Examples of atomic force microscopy images of α-syn, Aβ and tau oligomers are shown in Figure 2 (lower panel).

In addition to the dynamics underlying a transfer between different energetic states, environmental factors are believed to influence protein aggregation. Low pH, metal ions, certain lipids and the presence of various molecules, such as dopamine (Cappai et al., 2005), catechins (Ehrnhoefer et al., 2008), flavonoids (Hong et al., 2008) and oxidated methionine (Zhou et al., 2010) have all been suggested to influence the aggregation process. Some factors, such as oxidative stress, have been shown to act as triggers for oligomerization. For example, the reactive aldehydes 4-hydroxy-2-nonenal (HNE) and 4-oxo-2-nonenal (ONE) can promote oligomer formation *in vitro* by their covalent modification of α-syn monomers (Näsström et al., 2009; Nasstrom et al., 2011). Such reactions also occur in living cells as it was demonstrated that the addition of HNE results in the formation of α-syn oligomers that can propagate between neuroblastoma cells in culture (Bae et al., 2013).

Molecular factors can also influence protein aggregation kinetics. Post-translational modifications (PTMs) such as acetylation, arginylation, O-GlcNAcylation and other forms of glycosylation, phosphorylation, SUMOylation, and amino (N-) and C-terminal truncations play a critical role in aggregation and amyloid formation. Over 600 distinct PTMs have been experimentally identified, some of which have been proposed to correlate with the progression of neurodegenerative diseases (Schaffert and Carter, 2020). Some PTMs have been described to enhance or correlate with oligomer formation. Enzymatic O-GlcNAcylation of α-syn was shown to lead to an increased production of soluble oligomers while inhibiting its aggregation *in vitro* (Zhang et al., 2017). With respect to Aβ, phosphorylation of Ser26 was found to result in a stabilization of oligomers which do not aggregate further into insoluble fibrils. Moreover, pSer26Aβ oligomers showed increased toxicity, compared to other Aβ variants, in human neurons (Kumar et al., 2016). In several early-affected brain regions of AD patients, a signature of three tau PTMs was found to correlate with tau oligomerization (Ercan-Herbst et al., 2019). When acetylated at lysine 280, tau was found to form predominantly globular oligomers and short fibrils (<200 nm) *in vitro*, with a reduced propensity to aggregate into longer filaments compared to unmodified tau (Haj-Yahya and Lashuel, 2018). In Huntington’s disease, the intracellular accumulation of soluble toxic Htt oligomers may result from SUMOylation at lysine residues (Steffan et al., 2004). The study of the aggregation-modulating PTMs could elucidate potential therapeutic alternatives.

Interestingly, certain disease-causing mutations have been found to increase oligomer formation of pathogenic proteins. For example, the rare A53T and A30P *SNCA* mutations, which segregate with disease in Greek and Italian PD families, respectively, were both found to result in α-syn forms that are more prone to form large oligomers/protofibrils (Singleton et al., 2003; Kasten and Klein, 2013; Flagmeier et al., 2016; Trinh et al., 2018; Ohgita et al., 2022). Similarly, the early-onset AD *Arctic* mutation (E693G) in *APP* was shown to result in an increased formation of oligomeric/protofibrillar Aβ species (Nilsberth et al., 2001; Johansson et al., 2007). Several mutations in *MAPT*, linked to FTD with parkinsonism, were also found to increase tau oligomer formation (Maeda et al., 2018).

## 3 Toxicity of soluble oligomers

Several investigations have demonstrated that larger oligomers are more toxic to cells than insoluble aggregates *per se* (Figure 1B). The first observation suggesting that intermediately sized protein aggregates may be causing neurodegeneration came from the identification of the A53T *SNCA* mutation (Polymeropoulos et al., 1997), which leads to increased production of large (>600 kDa) spherical α-syn oligomers/protofibrils with a diameter of ~20 nm (Conway et al., 2000; Rochet et al., 2000). Subsequent findings from cell-based studies have suggested that *in vitro*-generated oligomers/protofibrils of α-syn can compromise cell membrane integrity (Volles and Lansbury, 2002; Danzer et al., 2007), impair synaptic excitability (Diogenes et al., 2012; Choi et al., 2013; Kaufmann et al., 2016), induce oxidative stress (Cremades et al., 2012), disturb mitochondrial function (Kaufmann et al., 2016) and decrease cell viability (Nasstrom et al., 2011).

Similarly, several studies have highlighted that oligomers/protofibrils of Aβ are toxic both *ex vivo* and *in vivo*. For example, Aβ oligomers (8–12 kDa) derived from APP transfected cells (Walsh et al., 2002) or extracted from human brain (Wang et al., 2017) were found to cause synaptic impairments on acute mouse hippocampal slices. *In vivo*, mice injected with Aβ oligomers secreted by human APP transfected cells display disrupted cognitive function (Cleary et al., 2005). Moreover, Shankar and colleagues showed that TBS-soluble Aβ dimers (8 kDa), but neither insoluble fibrils nor high molecular weight (HMW) soluble oligomers (>60 kDa) extracted from AD brain, could alter hippocampal synapse physiology and negatively impact learned behavior (Shankar et al., 2008). The elucidation of the *Arctic APP* mutation (Nilsberth et al., 2001) and the realization that the Arctic Aβ mutant promotes the formation of oligomers/protofibrils (Johansson et al., 2007) have further highlighted the central role of such species in the pathogenesis of neurodegenerative disorders. Most of the reported Aβ oligomer toxicity mechanisms relate to their interaction with and disruption of cell membranes (reviewed in Reiss et al., 2018).

Although hyperphosphorylated NFTs are referred to as the pathological hallmark in tauopathies and related disorders, the pathogenic effects of the insoluble NFTs have been debated (Terry, 2000; Wittmann et al., 2001; Spires-Jones et al., 2011). Instead, oligomers have been suggested to be the most neurotoxic tau species (Fox et al., 2011; Usenovic et al., 2015; Ozcelik et al., 2016). Subcortical injection of tau oligomers in wild type mice leads to memory impairment as well as synaptic and mitochondrial disruption (Lasagna-Reeves et al., 2011). Several intraneuronal processes, such as heterochromatin organization, mitochondrial function, synaptic plasticity, microtubule assembly and axonal transport, appear to be negatively affected by tau oligomers (reviewed in Niewiadomska et al., 2021). Mounting evidence also suggests that oligomers can directly impair proteasomal function. Thibaudeau et al. reported that soluble oligomers of Aβ1-42, α-syn, and Htt, but not monomers or mature fibrils, blocked the UPS (Thibaudeau et al., 2018). In addition, the toxic role of soluble Htt and TDP-43 oligomers in HD, FTLD and ALS research has also begun to be studied (Takahashi et al., 2008; Lajoie and Snapp, 2010; Legleiter et al., 2010; Fang et al., 2014).

Once an amyloidogenic protein has adopted a pathological conformation, that molecule could act as a seed for other proteins to adopt the same toxic conformation and initiate a spreading cascade. Via such a nucleating mechanism, the resulting protein species could in themselves become toxic to cells, but also act as building blocks in the formation of larger aggregates and fibrils. Whereas the intermediate sized species are believed to be particularly harmful, also the fibrils may cause damage by occupying intra- or extracellular space. The size of such assemblies may vary, as both oligomers and fibrils can have seeding capacity.

As yet another implication of this process, the pathological species may spread between cells and cause pathology in interconnected brain areas. As for the cell types involved, both neurons and astrocytes have been shown to transfer aggregates of α-syn and Aβ in cell models (Nath et al., 2012; Loria et al., 2017; Rostami et al., 2017). However, whether both types of cells are involved in protein propagation within the living brain remains unknown.

## 4 Seeding and propagation of amyloidogenic proteins

Accumulating evidence suggests that several of the proteins involved in neurodegenerative disorders possess “prion-like” seeding and propagation properties. According to this hypothesis, the pathogenic protein forms could transfer their toxic conformation to the physiological species, leading to disease propagation throughout the brain.

### 4.1 Seeding

As described above, seeding is a process initiated by the presence of a pathological conformer (the seed), which will serve as a driver for the aggregation process. Once a normally folded monomer (the template) has been in close contact with an abnormally folded species, it will itself adopt the same pathological conformation and become capable of inducing further conversion of proteins from their physiological to a pathological state (Morales et al., 2013). In the initial phase of this process, a nucleating protein species induces the conversion of soluble protein monomer to misfold and eventually form insoluble, higher-order assemblies (Xue et al., 2008; Greenwald and Riek, 2010; Xue, 2015). This mechanism of aggregation, which was first demonstrated for the prion protein as a central event in the pathogenesis of CJD as well as other prion disorders (Come et al., 1993), has now also been widely attributed to other misfolded proteins that cause neurodegenerative diseases (Cerda-Costa et al., 2007; Soto and Pritzkow, 2018). This understanding has led to the modification of *in vitro* prion aggregation assays to study the seeding capacity of other disease-related proteins.

Seed amplification assays (SAA) broadly describe various applications, such as real time quaking induced conversion (RT-QuIC) and protein misfolding cyclic amplification (PMCA) that take advantage of the capacity of a nucleating protein to seed a pathogenic conformation. The various SAAs can detect protein seeds at femtogram or attogram amounts and demonstrate high sensitivity in distinguishing disease biospecimens between controls and overlapping disease pathologies (Manca and Kraus, 2020). The conversion and aggregation rates depend upon the relative amounts of seed and template and the nature of the seed (i.e., WT or mutant). Additionally, seeding properties of nucleating particles can be studied in cell-based assays. Fluorescence resonance energy transfer (FRET) and other fluorescence-based readouts are employed to assess aggregation of proteins fused to fluorescent tags when exposed to biospecimen seeds (Holmes et al., 2014; Woerman et al., 2015).

Seeding effects can occur either between proteins of the same type or between different amyloidogenic proteins, in a process called cross-seeding. An example of homologous cross-seeding occurs with the *SNCA* G51D α-syn mutant. *In vitro* studies demonstrate that this heterozygous mutation results in an α-syn fibril that can cross-seed WT α-syn monomers into the same unique fibrillar structure as the mutant, as confirmed by transmission electron microscopy (TEM). Notably, the G51D α-syn fibrils induce significantly increased cytotoxicity in cell models compared to WT α-syn (Sun et al., 2021).

Similarly, also Aβ can undergo homologous cross-seeding. Point mutations that cause familial AD (FAD) are autosomal dominant, leading to heterozygous inheritance. Thus, in the affected brains an equal amount of WT Aβ, such as Aβ40 and Aβ42, can be assumed to be produced to the same degree as the mutated forms. Several studies have thus investigated how different variants and isoforms of Aβ may influence the amyloidogenic properties of the other forms. For example, a mixture of WT Aβ with the Aβ40 Arctic (E22G) mutant was found to promote Arctic Aβ driven fibril formation by an eight-fold reduction of the lag time in a Thioflavin T (ThT) aggregation assay. The specific differences observed in these interactions are believed to be governed by the compatibility of the topographical structure of the resultant fibrils (Liang et al., 2022).

Examples of heterogeneous cross-seeding have been described for several neurodegenerative proteins. By using an anti-oligomer antibody, it was found that Aβ may form oligomers together with both PrP, TDP43, and α-syn (Guerrero-Munoz et al., 2014). Yet another study found evidence that Aβ and α-syn can form seeding-competent hybrid oligomers with each other (Ono et al., 2012). Thus, such a molecular interaction may explain the increased incidence of AD brain pathology in PD (Boller et al., 1980). Moreover, in the Lewy body (LB) variant of AD, α-syn pathology coexists with Aβ pathology primarily in the amygdala. Taken together, more than half of sporadic and familial AD display Lewy body pathology (Kotzbauer et al., 2001; Vaikath et al., 2019). It has also been reported that Aβ and tau, the two proteins that always aggregate in the AD brain, can bind to each other, which could provide a molecular link between the two pathologies. However, it is unknown whether oligomers of these proteins are more prone to such interactions or whether the molecular interactions can also occur between physiological monomers (Guo et al., 2006).

### 4.2 Propagation of protein pathology

Similar to what was first described for PrP, other seeding-competent amyloidogenic proteins can be released and propagate beyond the location where they were formed. This phenomenon, known as spreading, occurs when such species are released from the cell of origin and taken up by neighboring cells, where they instigate further misfolding and polymerization. Accumulated seeds can be secreted directly into the extracellular space, transferred by extracellular vesicles, or transferred via cell-to-cell contact (reviewed in Peng et al., 2020). One of the first indications that amyloidogenic proteins can spread and propagate in the brain was the finding of Lewy body-like pathology in long-term grafted dopaminergic neurons in PD patients. These subjects had been part of a clinical trial aimed to mitigate the loss of striatal dopamine via cell transplantation (Kordower et al., 2008; Li et al., 2008; Mendez et al., 2008). This observation indicated that pathological proteins most likely had transferred from the affected host to the grafted tissue, supporting the hypothesis that pathological α-syn can propagate between interconnected regions and thereby explain the hierarchical pattern by which Lewy pathology appears in the PD CNS (Braak et al., 2003; Fahn, 2003).

A hierarchical spatial distribution of deposits has been described also for other amyloidogenic proteins, including tau and Aβ. As for PD brain pathology, plaques and tangles accumulate in topographically distinct patterns in the AD brain (Braak and Braak, 1991; Thal and Fandrich, 2015). The stepwise appearance in various interconnected brain areas suggests that pathological Aβ and tau can spread within the diseased brain. Cell-to-cell transport via exosomes has been described as a key mechanism for protein spreading in neurodegenerative diseases (Howitt and Hill, 2016). Misfolded tau seeds have been proposed to propagate trans-synaptically, moving between functionally connected neurons via exosomes, but also through vesicle-free mechanisms (Polanco and Gotz, 2022), while elevated Aβ oligomer levels have been found in exosomes isolated from AD brains (Sardar Sinha et al., 2018). Additionally, intercellular transport by tunneling nanotubes was reported in several neuronal cell lines for both tau and Aβ (Zhang et al., 2021). To add to the complexity, known disease-associated mutations could differentially regulate cell-to-cell uptake of amyloidogenic proteins. For example, the E46K mutation, as well as six other *SNCA* mutations, were found to enhance secretion and uptake of α-syn fibrils by cells through a calcium-dependent exocytosis pathway (Guan et al., 2020).

Experimental evidence from studies on transgenic mouse models strongly supports the hypothesis that these proteins can propagate between anatomically related regions (de Calignon et al., 2012; Liu et al., 2012). Several *in vivo* experiments have demonstrated how intracerebral injections of human-derived α-syn, Aβ and tau species can cause seeding and spreading of disease pathology in transgenic mice (Kane et al., 2000; Morales et al., 2012; Stohr et al., 2012; Watts et al., 2014; Lau et al., 2023; Mate De Gerando et al., 2023; Weber et al., 2023). Interestingly, peripheral inoculation of brain-derived Aβ and tau aggregates could also induce the formation of cerebral deposits (Eisele et al., 2010; Clavaguera et al., 2014); further supporting the hypothesis that PrP is not the only neurodegenerative disease-associated protein capable of propagating from the periphery to the brain.

## 5 Seeding and propagation of oligomers/protofibrils

Most of the above-mentioned experiments were carried out using fibrillar protein aggregates or preparations likely containing a heterogeneous mixture of protein assemblies with a range of sizes and solubility, making it difficult to speculate which species could be mediating the seeding. Regardless, differently sized oligomeric species might be key players in the seeding and propagating pathologies in neurodegenerative diseases. In the following, we will describe the most important findings related to the role of oligomeric protein assemblies in this context (summarized in Table 1).

**Table 1:** Seeding and spreading of amyloidogenic oligomers.

Protein	Main related diseases	Seeding and spreading of oligomers			Therapies targeting oligomers
		*In vitro* / SAA	Cell models	*In vivo*	
α-syn	PD, MSA, DLB	Iljina et al. (2016); Lau et al. (2022)	Danzer et al. (2007); Danzer et al. (2009); Hansen et al. (2011); Fagerqvist et al. (2013b); Illes-Toth et al. (2015); Iljina et al. (2016); Fusco et al. (2017); Cascella et al. (2021)	Boi et al. (2020)	BAN0805, phase I (Nordstrom et al., 2021) Minzasolmin, phase II (Price et al., 2023) Emrusolmin, phase II (Heras-Garvin et al., 2019)
Aβ	AD	Salvadores et al. (2014); Chang et al. (2022)	Olsson et al. (2018)	Langer et al. (2011); Stohr et al. (2012); Katzmarski et al. (2019)	Lecanemab (Leqembi, approved by FDA for treatment of AD) (van Dyck et al., 2023) ALZ-801, phase III (Alzheon, 2021) PRI-002, phase I (Zhang et al., 2019)
Tau	AD, FTD, PSP, CBD	Lasagna-Reeves et al. (2012); Ghag et al. (2018)	Ruan et al. (2021); Mate De Gerando et al. (2023)	de Calignon et al. (2012); Liu et al. (2012); Lasagna-Reeves et al. (2012); Mate De Gerando et al. (2023)	APN-005, phase I (Hwan-Ching et al., 2022) OLX-07010, phase I (Tian et al., 2013) TRx0237 (LMTX™), phase III (Wischik et al., 2022)
PrP^Sc^	TSEs	Silveira et al. (2005); Kim et al. (2012)	–	–	–
Htt	HD	Morozova et al. (2015)	Herrera et al. (2011); Cicchetti et al. (2014); Pecho-Vrieseling et al. (2014); Tan et al. (2015)	–	–
TDP-43	FTLD, ALS	–	Montalbano et al. (2020)	–	–

### 5.1 Alpha-synuclein

One of the pioneering studies investigating the spreading and seeding of α-syn oligomers was conducted by Karin Danzer and colleagues in 2007. By incubating recombinant α-syn with or without FeCl_3_, different oligomers were generated. The group characterized the generated oligomers by atomic force microscopy (AFM) analyses and described heterogeneous populations of globular and protofibrillar structures ranging from 2 to 23 nm in height, depending on the preparation. When subjecting cultured neuroblastoma cells to fluorescently labeled versions of such protein species, it was observed that some of them could cause pores in the cell membrane, whereas others instead were efficiently internalized by the cells and induced co-aggregation with either overexpressed or endogenous human α-syn (Danzer et al., 2007). In a follow-up study, the research team demonstrated that this type of transmembranous seeding by α-syn oligomers can occur in several cell types, including primary neurons (Danzer et al., 2009).

In another study, Illes-Toth and colleagues generated differently sized and configured oligomers *in vitro* by co-incubating α-syn with either ammonium acetate and 20% ethanol or with pure ethanol (Illes-Toth et al., 2015). These species were characterized by mass spectrometry (MS) and found to range from unordered dimers to compact large oligomers. Similar to the findings in Danzer et al., the authors identified two major classes of oligomers, pore-forming species that lack seeding properties and higher-order seeding competent oligomers (Illes-Toth et al., 2015).

Central features of these studies were replicated in yet other investigations. In a study by Hansen et al., using a co-culture system, it was reported that different forms of α-syn, including oligomers, can be taken up by human embryonic kidney (HEK) cells and then propagate to neuroblastoma cells where they interact with intracellular α-syn (Hansen et al., 2011). In a study from our group, we found that large oligomers (∼2000 kDa), generated by incubating recombinant human wild-type α-syn with the reactive aldehyde ONE, are efficiently taken up by cultured neuroglioma cells and that they can induce seeding *in vitro* (Fagerqvist et al., 2013b).

In another study, both *in vitro* and cell-based seeding conditions were explored (Iljina et al., 2016). The authors applied single-molecule FRET to define two classes of oligomers, those with a disordered “low FRET” conformation and those with a compact “high FRET” conformation. In the test tube, both fibrils and oligomers were found to efficiently seed α-syn monomers. However, under cell culture conditions, it was observed that the oligomers can cause seeding and cytotoxicity at lower concentrations than fibrils. The authors suggest that differences in their ability to induce cellular oxidative stress may explain why oligomers can cause a more aggravated seeding reaction than fibrillary species of α-syn (Iljina et al., 2016).

In our previous study, we also investigated if α-syn oligomers can induce seeding *in vivo*. Thirteen-month-old homozygous male and female (Thy-1)-h[A30P] aSYN transgenic mice were injected intracerebrally with either 40 ng or 400 ng of ONE-induced human wild-type α-syn oligomers, but no consistent pattern of aggravated pathology could be observed in the vicinity of the injected material (Fagerqvist et al., 2013b). In a more recent study, *in vitro* generated α-syn oligomers (650–1,100 kDa) were injected into the substantia nigra of living rats, and increased pS129-α-syn deposition could be detected in both the injected area and the striatum (Boi et al., 2020). In addition, a gradual nigrostriatal dopaminergic loss associated with motor and cognitive impairment was observed in the injected rats. The oligomers used in this study have been shown to disrupt lipid bilayers *ex vivo*, cause cellular toxicity, induce reactive oxygen species, and reduce mitochondrial activity in neurons (Fusco et al., 2017). Structurally, they displayed several interesting features; an accessible N-terminal region, an exposed highly lipophilic region that can promote interaction with the surface of cellular membranes, and a rigid oligomeric core rich in β-sheet structures that can insert into the lipid bilayer and disrupt membrane integrity. These features may explain the high toxicity of such α-syn oligomers and why they displayed such pronounced seeding properties (Fusco et al., 2017).

More recently, it was shown that α-syn fibrils can release α-syn oligomers *in vitro* (Cascella et al., 2021). Based on these findings, the authors suggested that, in addition to contributing to toxicity, such oligomeric species can potentially enhance the generation of new aggregates and further contribute to pathogenesis via neuron-to-neuron spreading or by generating new fibrils. Another *in vitro* study compared the seeding potential of purified recombinant α-syn oligomers and preformed fibrils. Although oligomers were seeding competent, sonicated preformed fibrils seeded more efficiently (Lau et al., 2022). The authors suggested that such differences may be explained by the amplification process. Oligomers might first need to refold into ThT-positive species to become structurally more β-sheet enriched, while fibrils amplify by elongating (Lau et al., 2022). Such findings also highlight the importance of having robust oligomer purification methods, as even a low amount of fibrils could impact seeding in the applied assays.

Looking into *post mortem* brains, a different distribution between soluble α-syn oligomers, identified using a proximity-ligation assay (PLA) on paraffin-embedded sections, and insoluble LB aggregates was described in brains of PD patients, suggesting that oligomers might be widespread at an early disease stage and act as a substrate for Lewy body inclusion as the disease progresses (Sekiya et al., 2022). Earlier affected regions appear to present abundant LB pathology, but lower oligomer levels than newly affected regions. Interestingly, a hierarchical α-syn seeding activity in SAA was observed in PBS soluble brain extract of Lewy body disease (LBD) patients, with the highly affected substantia nigra region showing low seeding activity compared to later affected structures, such as the amygdala and hippocampus (Martinez-Valbuena et al., 2022a).

### 5.2 Amyloid-β

Similar to α-syn, Aβ oligomers have also been shown to play a central role in seeding and propagation of pathology. Initial studies found that exogenously administered forms of either synthetic or brain-derived Aβ aggregates can result in aggravated pathology in *APP* transgenic and knock-in mice (Meyer-Luehmann et al., 2006; Stohr et al., 2012; Ruiz-Riquelme et al., 2018). Subsequent studies found that several different isoforms of synthetic Aβ can generate seeding-competent assemblies (Stohr et al., 2014; Ruiz-Riquelme et al., 2021). While these studies revealed “prion-like” properties of Aβ aggregates, they did not identify which Aβ species are responsible for the seeding behavior.

Soluble and proteinase K (PK) sensitive Aβ preparations, extracted from APP23 transgenic mice, were also found to induce Aβ deposition when injected intracerebrally into young APP23 mice (Langer et al., 2011). Compared to the deposition generated by the totality of Aβ present in the mouse brain extract, soluble Aβ species found in the supernatants of brain homogenates after 100,000x *g* centrifugation, were responsible for 30% of the β-amyloid load in injected mice, despite accounting for less than 0.05% of the total Aβ in the unprocessed homogenate. These findings suggest that these soluble Aβ forms possess very high seeding activities, which was supported by the fact that sonication of the Aβ containing homogenates led to an increase in soluble Aβ levels and seeding activity (Langer et al., 2011). Interestingly, while highly potent PBS-soluble Aβ seeds could also be isolated from AD brains, CSF from AD patients did not seed Aβ deposition in transgenic mice, despite overall higher Aβ levels in such samples, but remained seeding-competent in an *in vitro* setting (Fritschi et al., 2014).

To better understand the contribution of Aβ oligomers in disease pathogenesis, a study investigated how the occurrence and propagation of pathology is affected by different Aβ species (Katzmarski et al., 2019). Young *APP* transgenic mouse brains were inoculated with brain homogenates from aged mice, and by immunodepleting such samples with different Aβ antibodies it could be concluded that oligomers were particularly potent in terms of inducing pathology. However, no significant seeding effects took place when instead brain extracts or CSF from younger brains were injected (Katzmarski et al., 2019). Thus, Aβ oligomers might not be capable of seeding by themselves as they seem to require the presence of either other Aβ species or unknown molecular factors.

Employing reverse micelles to prepare uniformly sized HMW oligomeric Aβ (>650 kDa), Chang and coworkers showed by site specific NMR that Aβ1-42 oligomers can promote aggregation and fibrillization of Aβ1-40 monomers *in vitro*, and modulating the conformation of the resulting Aβ1-40 oligomers (Chang et al., 2022). *Ex vivo*, oligomeric intracellular inclusion (250–670 kDa) extracted from N2a neuroblastoma cell lines after treatment with transgenic AD mouse brain extracts were found to induce inclusion formation in naive APP-expressing cell lines, suggesting that Aβ oligomers are capable of inducing seeded nucleation in a cellular model (Olsson et al., 2018).

### 5.3 Tau

The tau protein has also been found to propagate between cells, both in culture (Ruan et al., 2021) and in the living mouse brain (de Calignon et al., 2012; Liu et al., 2012). As for the tau species involved, emerging evidence suggest that the oligomers are particularly prone to cause seeding reactions and promote cell-to-cell spreading.

Tau oligomers isolated from PBS soluble AD brain extracts were found to induce aggregation of monomeric recombinant human tau *in vitro* (Lasagna-Reeves et al., 2012). Such oligomers were characterized by AFM as well as immunoblotting and corresponded to tau dimers/trimers with a MW of 110–160 kDa. In the same study, seeding effects of the extracted tau oligomers on endogenous tau were evaluated in wild-type mice upon hippocampal injection. It was found that tau oligomers can induce widespread tau pathology in the hippocampus and adjacent areas, such as cortex, corpus callosum and hypothalamus, 11 months post injection. These observations could not be made in mice injected with insoluble paired helical filament tau, indicating that tau oligomers, but not fibrils, are able to seed and induce tau propagation in this experimental model (Lasagna-Reeves et al., 2012). In a study by Ghag and colleagues, AFM-based investigations showed that tau oligomers made from sonicated samples of recombinant tau could readily convert added monomers into similar oligomers with an average height of 3–5 nm. Moreover, the authors evaluated cell viability and concluded that the generated oligomers were the most toxic tau species (Ghag et al., 2018). In a separate study, the same research group investigated the potential cross-talk between α-syn and tau (Castillo-Carranza et al., 2018). Upon subjecting cultured tau-overexpressing cells to recombinant α-syn seeds, toxic tau oligomers were formed. Moreover, when inoculating PD-derived α-syn oligomers into the brain of tg *MAPT* mice, the formation of toxic tau oligomers was accelerated (Castillo-Carranza et al., 2018). In another study, the seeding abilities of brain-derived tau oligomers and fibrils were compared both *in vitro* and *in vivo*. High molecular weight tau oligomers (400–600 kDa), isolated from AD brain via size exclusion chromatography, showed a seeding potency equal to insoluble tau PHF in a cell bioactivity assay, as well as a similar neuronal uptake in the hippocampus of transgenic mice (Mate De Gerando et al., 2023). The same study showed that tau oligomers might induce a quicker propagation of misfolded tau across anatomically connected regions and cause a higher glial activation than insoluble tau filaments.

Interestingly, Mirbaha and coworkers reported that tau monomers can be seeding competent when possessing a specific conformation. The authors used size exclusion chromatography to isolate recombinant fibril-derived monomers, which exhibited cellular and *in vitro* seeding activity (Mirbaha et al., 2018). The structure of the seeding competent monomers was characterized by cross-linking with mass spectrometry (XL-MS) and was found to differ from that of inert tau monomers. Seeding competent tau monomers were also isolated from AD brains but were not found in healthy controls.

### 5.4 PrP^Sc^

Although PrP is not the main topic of our review, we also want to highlight the most recent findings on oligomeric PrP^Sc^ seeding and spreading. While the infectivity of mammal prion diseases was originally attributed to proteinase-resistant PrP^Sc^ deposits (Prusiner, 1982; Prusiner et al., 1983), the lack of such species in an increasing number of diseases (Gambetti et al., 2008; Colby et al., 2010) led investigators to question whether smaller and more protease-sensitive forms of PrP^Sc^ could be responsible for the initial step or alternative misfolding of physiological PrP (Bishop et al., 2010; Kim et al., 2011; Miyazawa et al., 2011). As with proteins involved in other neurodegenerative disorders, oligomers of recombinant PrP were more toxic than their fibrillar counterparts (Simoneau et al., 2007), although as a caveat of this observation recombinant PrP adopts aggregate structures that are fundamentally distinct from infectious PrP^Sc^ present in brain (Wille et al., 2009; Kraus et al., 2021; Manka et al., 2022). Silveira et al. were able to correlate infectivity of hamster 263 K PrP^Sc^ to its particle size, showing that non-fibrillar, oligomeric, assemblies between 14 and 28 PrP molecules (300–600 kDa) presented the highest infectious capability, while large fibrils were less infectious (Silveira et al., 2005). The study also showed no appreciable infectivity and converting activity when oligomers were formed by ≤5 PrP molecules. Other studies have supported the hypothesis of oligomeric PrP^Sc^ being potent seeders. Kim and co-workers investigated the presence and seeding activity of small aggregates of PrP^Sc^ in the brain of sporadic CJD patients. Ultracentrifugation of brain homogenates in a sucrose gradient showed a wide range of PrP^Sc^ aggregates size, from to <20 to >600 PrP^Sc^ molecules. Interestingly, the most potent seeders in QuIC and sPMCA assays were small oligomers of human PrP^Sc^ ranging between 20 and 78 molecules (Kim et al., 2012).

### 5.5 Other amyloidogenic proteins

In addition to α-syn, Aβ, and tau, other proteins known to accumulate in neurodegenerative conditions can also form oligomers that seed wild-type monomers of the same species and/or cross-seed other proteins. High molecular weight oligomers (>440 kD) of recombinant full-length human TDP-43 have been found capable of cross-seeding Aβ to form oligomers. Recombinant TDP-43 oligomers, isolated by size exclusion chromatography, were characterized by TEM and dynamic light scattering (DLS) and presented as a heterogeneous population of spherical or ring-shaped aggregates with a diameter between 40 to 400 nm. The interaction between TDP-43 and Aβ oligomers has been demonstrated both in the forebrain of transgenic TDP-43 mice and in brains of FTLD patients (Fang et al., 2014). Montalbano et al. confirmed that oligomeric TDP-43 is present in multiple neurodegenerative disorders and demonstrated cross-seeding capacity of TDP-43 oligomers and tau in HEK cells with human brain derived TDP-43. Treatment of cells with recombinant tau increased the relative amounts of TDP-43 oligomers in the nuclei and cytoplasm while overall amounts of TDP-43 were not significantly changed. These findings suggest that tau promotes the conversion of monomeric TDP-43 into oligomeric forms. Further, cross-seeding of tau aggregation was observed by TDP-43 oligomers isolated from AD, ALS and FTD brains by immunoprecipitation with an oligomer-selective anti-TDP-43 antibody (Montalbano et al., 2020).

Also for Htt, oligomeric species seem to be central for the spreading of pathology in the HD brain. Pathological expansions of the polyglutamate repeat regions of exon 1 of *HTT* can promote the formation and intercellular transfer of oligomers (Herrera et al., 2011; Cicchetti et al., 2014; Pecho-Vrieseling et al., 2014). Intriguingly, Htt oligomers isolated from affected brains were shown to potently seed monomeric Htt (Morozova et al., 2015). Moreover, it has been shown that patient and transgenic mouse CSF-derived mutant Htt can act as seeds for the continuous formation of Htt oligomers (Tan et al., 2015). For SOD1, no studies to date have clearly indicated that oligomers are formed as a part of the pathogenic cascade for ALS.

## 6 Molecular and environmental prerequisites for seeding and propagation of oligomers

As the generation of toxic and/or seeding competent oligomers is a gradual process, it is of importance to understand what features in the monomeric protein are governing its transition from a physiological to a pathological species (Figure 1). Thus, which molecular prerequisites need to be in place for the seeding and spreading of α-syn, Aβ, tau and other amyloidogenic proteins in the neurodegenerative brain? As exemplified above, oligomers of these proteins seem to be particularly prone to seeding and propagation. However, not all types of oligomers have such properties.

Firstly, size matters. As observed in the studies by Danzer et al. (Danzer et al., 2007) and Illes-Tothet al. (Illes-Toth et al., 2015), primarily larger α-syn oligomers (6–150 monomer units) are internalized and cause seeding in the recipient cells. The study by Iljina et al. utilized FRET measurements to characterize the α-syn oligomers (Iljina et al., 2016). By this method, several interesting observations could be made. In addition to the existence of one smaller (with less than 10 monomeric subunits) and two different larger oligomers (disordered “low-FRET” and ordered “high-FRET” oligomers) the authors were able to show that only the larger “high-FRET” oligomers can promote the generation of reactive oxygen species and thereby create an environment of oxidative stress. However, it was found both in this study and in the study by Danzer et al. that the disordered non-seeding competent oligomers can be transformed into highly ordered oligomers with seeding properties (Danzer et al., 2007; Iljina et al., 2016). Thus, several studies suggest that most α-syn oligomers are *en route* to fibril formation. Such on-pathway oligomers are thus believed to eventually become integrated into Lewy bodies and Lewy neurites in the affected brain. However, the existence of off-pathway α-syn (oligomeric species that do not aggregate further) species have also been identified and may play an important role in neurodegeneration due to their resistance to degradation. The seeding capacity of ONE (4-oxo-2-noneal) induced off-pathway α-syn oligomers (47 kDa) has been demonstrated *in vitro* (Fagerqvist et al., 2013b).

Similarly, the size of tau aggregates was found to affect their internalization and availability to seed intracellular aggregation (Mirbaha et al., 2015). *In vitro* studies have shown that aggregates (100-250 kDa) and short fibrils of tau bind to the surface of neurons with greater efficiency than monomeric or long fibrils of tau, followed by their internalization at the axon terminal or somatodendritic compartment via bulk-endocytosis (Guo and Lee, 2011; Wu et al., 2013). Internalized tau is subsequently trafficked to the lysosomes and released to the cytoplasm following dissolution of the vesicle wall (Wu et al., 2013; Calafate et al., 2016; Wegmann et al., 2016). A study found that HMW phosphorylated tau oligomers (>400 kDa) extracted from brain extracts of rTg4510 tau-transgenic mouse lines are more robustly taken up by neurons and, albeit being less abundant, have an increased seeding activity compared to low molecular weight tau species (Takeda et al., 2015).

Additionally, Aβ42 oligomers of different sizes prepared *in vitro* were found to aggregate at different rates in a ThT aggregation assay (Chen and Guo, 2023). Interestingly, the size of soluble Aβ aggregates was different between brain extract and CSF samples and differences in both *in vitro* and *in vivo* seeding activities were reported for the two preparations. Despite being at a 10-fold higher concentration than brain-derived homogenates, the smaller CSF-derived oligomers failed to seed Aβ aggregation in APP23 transgenic host mice, while appreciable amyloid-β deposition was observed 11 months post intrahippocampal injections of soluble AD brain fractions (Fritschi et al., 2014). The authors suggest that, because of their smaller size, Aβ oligomers in CSF samples might be too unstable to seed *in vivo.* Additional factors, e.g., the presence of N-terminally truncated Aβ species which are absent in CSF, might contribute to the seeding abilities of brain-derived preparations. Despite the lack of *in vivo* seeding activity, the authors reported that CSF-derived Aβ showed some seeding activity in an *in vitro* setting, supporting the findings of a previous study, indicating that oligomeric Aβ in AD CSF induces a seeding response, as measured by the protein misfolding cycle amplification assay (PMCA) (Salvadores et al., 2014). Interestingly, seeding activity in such CSF samples was higher compared to that of healthy controls (Salvadores et al., 2014). As previously mentioned, Aβ dimers have been linked with the development of memory impairment *in vivo* (Shankar et al., 2008). A recent study showed how Aβ-S8C dimers, stabilized by a disulfide bond, inhibit Aβ1-42 fibril formation *in vitro*, and lead to a reduction of Aβ plaques in TgCRND8 mice (van Gerresheim et al., 2021). Such studies highlight the importance of understanding the toxicity of on-pathway and off-pathway oligomers and raise the question of how well aggregation propensity and disease relevance correlate with each other.

Secondly, shape is important. Despite that different oligomerizing agents had been used in two previous studies on α-syn oligomer seeding (Danzer et al., 2007; Iljina et al., 2016), both studies indicated that the seeding competent oligomers are compact and have ring-like structures. Thus, by acquiring a certain size and structure the oligomer seems to become less amenable to enzymatic degradation and therefore stable enough to prevail and induce conformational changes of monomeric proteins within the cell (Danzer et al., 2007; Iljina et al., 2016).

Thirdly, it seems relevant which epitopes that are exposed on the outside of the oligomeric assemblies. In the work by Illes-Toth and colleagues the various *in vitro* generated α-syn oligomers were investigated by dot blot with a panel of monoclonal α-syn antibodies (Illes-Toth et al., 2015). It was found that antibodies against amino-terminal epitopes can only detect the pore-forming α-syn species and that antibodies against the mid-region also recognize the seeding-competent species. In contrast, carboxy-terminal antibodies have affinity only against seeding-competent α-syn. Interestingly, the A11 antibody, selective for oligomeric assemblies of many different proteins, including α-syn, was found to only bind the pore-forming species and not the species that cause seeding. Taken together, the authors speculate that the mid region and carboxy-terminal half of α-syn constitute conformational hotspots of the protein. By forming a ring-like structure in which these regions are exposed, α-syn adopts a higher-order configuration needed for seeding and intracellular aggregation (Illes-Toth et al., 2015).

It could be speculated that the extent of oligomerization needs to reach a threshold before a pathologically relevant degree of seeding can occur. Mainly, it is believed that a certain number of oligomers needs to be present for the seeding reaction to take place. The study by Iljina et al. found evidence of seeding and cell toxicity of oligomeric α-syn at submicromolar to low micromolar concentrations and estimated the corresponding lower threshold to be approximately 104 oligomers/cell (Iljina et al., 2016).The extent of cellular organelles and lipid surfaces (Galvagnion et al., 2015), alterations in salt content or pH (Buell et al., 2014), presence of molecular chaperones (Daturpalli et al., 2013) and varying efficiencies in protein degradation systems (Hao et al., 2013) could all provide triggers for seeding and propagation. In particular, the ubiquitin proteasome system (UPS) plays a key role in cellular quality control via targeted protein degradation. Under pathological conditions, the UPS and other protein quality control elements ultimately fail, allowing for the persistence of toxic oligomers and aggregated species. The accumulation of ubiquitinated aggregated proteins is a hallmark of many neurodegenerative disorders (Wang et al., 2008; Thibaudeau et al., 2018; McKinnon et al., 2020; Wesseling et al., 2020). These features could explain differences in the cellular content of oligomers and thereby contribute to seeding and propagation.

## 7 Strains

As a main feature of prion disorders, various protein aggregate conformations (“strains”) can act as templates for the generation of new pathogenic species. Such strains have certain disease characteristic properties but can also show case-to-case variations. For PrP, many strains have been categorized based on differences in their biochemical and neuropathological properties as well as their propagation behavior upon injection into mice. In general, mice that have been inoculated with unstable PrP^Sc^ strains will display pathology faster than mice that have been given a PrP^Sc^ strain with a more stable conformation (reviewed in Prusiner, 2013). The distinct types of disease brain derived protein filaments as discovered by cryo-electron microscopy (cryo-EM) lend further support to the strain hypothesis. Indeed, recent analyses of brain derived fibrils showed differently shaped filaments across the neurodegenerative diseases. A recent study on Aβ42 revealed high-resolution structures of two predominant Aβ42 assembly types. Type 1 predominates in sporadic AD and consists of paired Aβ42 filaments closely cradled in an “S” shape with amino-terminal protrusions from each “S,” whereas type II predominates in familial AD and diseases with parallel AD pathology. Type II structures are similar to those of type I insofar that they also consist of two Aβ42 filaments curved in an “S” shape, although they are not closely stacked together and lack the amino-terminal extensions observed in type I (Yang et al., 2022a). Similarly, cryo-EM structures of brain derived α-syn from PD, PDD, and DLB cases have been described, mainly as a single predominant fibril structure with a right-handed twist and a “Lewy-fold” filament core. In contrast, α-syn fibrils from MSA brain have been shown to have a left-handed twist (Schweighauser et al., 2020; Yang et al., 2022b). Similarly, the structure of protease resistant TDP-43 filaments from ALS and FTD brains was described as a single protofilament composed of stacked TDP-43 molecules in a double-spiral fold (Arseni et al., 2022). Finally, it has been found that tau folds between individuals are similar within the same diagnosis but show variations between diseases ([Bibr ref62][Bibr ref55], 2019; Arakhamia et al., 2021; Shi et al., 2021).

Accumulating evidence suggests that the strain concept is of relevance also for the development of other neurodegenerative disorders. Previous studies have indicated that pathogenic α-syn can adopt different strain characteristics (Watts et al., 2013; Prusiner et al., 2015; So and Watts, 2023). When young transgenic α-syn mice were intracerebrally inoculated with brain homogenates from either MSA patients or from already diseased mice the neuropathological and behavioral features were accelerated, whereas inoculation with brain homogenates from healthy human brains did not affect the pathogenesis. Similar results were obtained when using recombinant α-syn strains generated by polymerizing the protein using different buffer conditions (Lau et al., 2020). Although these results imply that pathological α-syn can promote the conversion of regular α-syn in the recipient brains the exact nature of such species remains unknown.

Amyloid-β aggregates can also display specific strain-like behaviors (reviewed in Lau et al., 2021). In one study, synthetic versions of the two most prevalent forms of Aβ - with 40 (Aβ40) and 42 (Aβ42) amino acids - were prepared in a 10 mM sodium phosphate buffer, with or without the presence of 0.1% (wt/vol) SDS, and injected into brains of *APP* transgenic mice (Stohr et al., 2014). Analysis of the generated synthetic Aβ40 and Aβ42 fibrils with TEM revealed different structures between the variants, with long and straight fibrils in Aβ40 preparations while Aβ42 samples were composed of short fibrils. The presence of 0.1% SDS during polymerization abolished such differences. Interestingly, the two preparations caused different Aβ pathology composition and distribution when injected into the brains of *APP* transgenic mice, differences that were no longer present when fibrils were generated in the presence of SDS. These variations suggest that various Aβ species have different strain properties.

In another study, brains of transgenic *APP* mice were inoculated with homogenates from AD brains with or without different *APP* mutations as well as with non-pathological control brains. For example, when studying brains inoculated with samples from a brain with the *APP* Arctic mutation (E693G), it was found that the induced pathology resembled the pathology seen in the human brain (Watts et al., 2014). Most notably, the plaque pathology displayed protease resistance and Aβ deposited in the vessel walls had a furry appearance, similar to the deposition in humans. Intriguingly, these features were preserved when inoculating a second set of mice with brain homogenate from the first set of inoculated mice. As the inoculated mice only produce wild-type Aβ, this confirmed that the presence of distinct strains of Aβ aggregates is responsible for the variation in pathological phenotype, not simply differences in Aβ sequence (Watts et al., 2014). Thus, Arctic Aβ (E22G) seems to have certain properties that render it prone to seed and propagate as a characteristic strain within the brain. Moreover, the APP Dutch mutation (E22Q) generates an Aβ version that was also found to induce strain-specific features in the recipient mouse brain (Condello et al., 2018). Interestingly, when subjecting tissues to structure-sensitive amyloid-binding dyes, it could be seen that sporadic AD brains, i.e., where no mutations have been found, could also display highly individual Aβ conformations in their deposits (Condello et al., 2018). Thus, it seems as if Aβ can adopt individual structural signatures, which can not only be seen for the rare familial variants.

Tau can also display strain-like features (Clavaguera et al., 2009; Sanders et al., 2014; Woerman et al., 2016, 2017), which could be hypothesized to be dependent on the influence of Aβ. Interestingly, when analyzing the number of pathological conformers in a large number of AD brain samples, it was found that self-replicating competent tau species are more abundant in younger cases with a lower age at onset as compared to more aged brains (Aoyagi et al., 2019).

Different strains of TDP-43 have also been reported. A study reported that TDP-43 aggregates derived from brain with different FTLD subtypes had different seeding effects on cultured cells, maintaining characteristics of the original subtype (De Rossi et al., 2021). Moreover, distinct such TDP-43 strains showed different susceptibility to PK digestion, spreading pattern and they developed distinctive morphological aggregates when injected into the brain of transgenic mice (Porta et al., 2021).

Accumulating evidence suggests that oligomeric strains might be present in neurodegenerative diseases. Amyloid-β HMW aggregates isolated from aqueous extracts of AD brains were found to possess a fibrillary structure, comparable to insoluble fibrils derived from plaques (Stern et al., 2023), suggesting that such dispersed HMW Aβ structures could be on-pathway aggregates. Different strains of oligomeric assemblies might be leading to disease subtypes and inter-individual variability. For example, recent SAA-based studies on α-syn from PBS-soluble *post mortem* human brain fractions showed differences in seeding activity between different LBD subtypes and heterogeneity between different brain regions (Martinez-Valbuena et al., 2022a,b). The structural characterization of misfolded oligomeric assembly is challenging because of their tendency to form higher-order aggregates and the rapidity of such conversions. For the same reason, it is difficult to determine which oligomeric structures have the most pathogenic relevance.

## 8 Diagnostic and therapeutic applications

In general, the use of oligomeric species for diagnostic application is still underdeveloped. Several studies, based on traditional ELISAs, report that oligomeric α-syn (Tokuda et al., 2010; Hansson et al., 2014) and Aβ (Fukumoto et al., 2010; Herskovits et al., 2013) are increased in CSF of PD and AD patients, respectively (Salvadores et al., 2014). However, such differences have been small and therefore not diagnostically useful.

**Figure 3:**
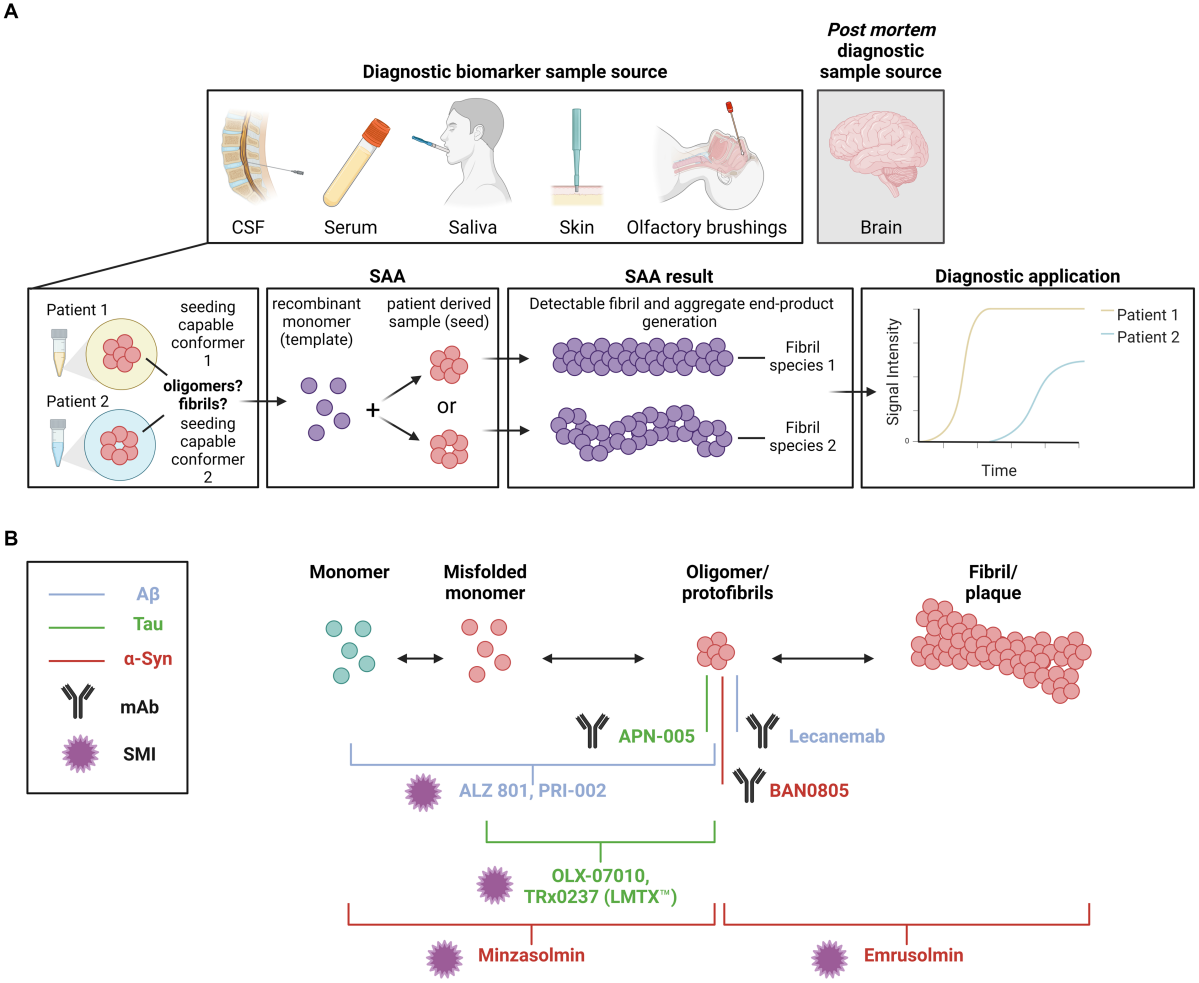
**(A)** Diagnostic applications targeting seeding capable species. The seeding amplification assay (SAA) has been proposed as a tool that can discriminate the seeding behavior of different protein species between diseases with overlapping pathologies and symptoms. Various sample sources, including CSF, serum, saliva and skin, can be used in the reactions. Analyses can also be conducted *post mortem* using brain tissue samples. Seeding of a recombinant monomer template by different oligomer species between samples can be visualized via Thioflavin T (ThT). **(B)** Therapeutic approaches targeting oligomers. Schematic representation of small molecule inhibitors (SMI) and monoclonal antibody (mAb) therapeutics in clinical trial. The primary target of each therapeutic is indicated by a line or bracket. Blue indicates therapeutics targeting Aβ. Green indicates therapeutics targeting tau. Yellow indicates therapeutics targeting α-syn. The antibody symbol indicates which therapeutics are monoclonal antibodies. The star indicates which therapeutics are SMIs. Created with BioRender.com.

Recently, SAA has been proposed as a diagnostic tool to discriminate between diseases with overlapping pathologies and symptoms, which are often misdiagnosed at early stages (for an overview of the underlying principles, see Figure 3A). Several retrospective and prospective studies using CSF RT-QuIC for the diagnosis of CJD showed high specificities (99–100%; Atarashi et al., 2011; Orru et al., 2015; Foutz et al., 2017; Fiorini et al., 2020; Rhoads et al., 2020) and the test has been implemented in several surveillance centers as a diagnostic tool for sCJD (Hermann et al., 2021). Excellent sensitivity in detecting PrP^Sc^ has been described when adopting PMCA on CSF (Barria et al., 2018), plasma (Bougard et al., 2016; Concha-Marambio et al., 2016) and urine (Moda et al., 2014). Using an assay based on seeding of α-syn, Fairfoul and colleagues reported a sensitivity of 92 and 95% and a specificity of 100% for correctly identifying DLB and PD, respectively, compared to AD and healthy controls in a small number of CSF samples (Fairfoul et al., 2016). When analyzing a somewhat larger set of CSF samples, cases with and without α-syn disorders were differentiated with a sensitivity of 75% and a specificity of 94% (van Rumund et al., 2019). Similarly, SAA on CSF samples was successfully used to discriminate between PD and MSA α-syn strains. A study from Claudio Soto’s group showed that such an assay could differentiate between these two pathologies with 95.4% sensitivity (Shahnawaz et al., 2020). Efforts are being made to develop SAA diagnostic tools for these pathologies using samples whose collection is less invasive than obtaining CSF, such as serum (Okuzumi et al., 2023), skin biopsies (Wang et al., 2020; Martinez-Valbuena et al., 2022c) and saliva (Luan et al., 2022). In addition, SAA could be used to discriminate between different tauopathies in relation to the tau repeat mostly present in the respective disease (Saijo et al., 2020). With PMCA, seeding competent Aβ oligomers were detected to a larger extent in AD CSF as compared to CSF from healthy controls (Salvadores et al., 2014). As such soluble species seem to be pathogenic in several neurodegenerative diseases, there is a rationale for implementing them as diagnostic biomarkers. The analyzed biospecimens are likely to contain several species (monomers, differently sized oligomers, protofibrils, fibrils) of the pathogenic protein. Despite the rapid advancement of diagnostic SAAs, the contribution of the different species is understudied and at present unclear. Comparative studies in the biomarker field would be essential to determine the relevance of the different species and could lead to more specific and sensitive diagnostic tools.

The realization that oligomers or protofibrils of certain proteins are central to the pathogenesis of neurodegenerative diseases has also offered novel therapeutic opportunities (summarized in Table 1 and illustrated in Figure 3B). In collaboration with the Swedish biotech company BioArctic AB, we generated monoclonal antibodies against toxic oligomers/protofibrils of α-syn (Fagerqvist et al., 2013a) and showed that they can be applied for passive immunotherapy to decrease pathology in a cell model and in the central nervous system (CNS) of transgenic mice (Lindstrom et al., 2014). One of these antibodies has been humanized as BAN0805, or ABBV-0805, and completed a phase I trial for disease-modifying treatment of PD (Nordstrom et al., 2021).

A monoclonal antibody against Aβ oligomers/protofibrils, mAb158, has also been developed by the Uppsala group and shown to influence brain pathology upon peripheral administration in a mouse model (Lord et al., 2009). A humanized version of mAb158, Lecanemab, slowed cognitive decline, reduced accumulation of amyloid plaques, as measured by PET, and improved biochemical parameters in cerebrospinal fluid (CSF) of AD patients in both a phase IIb (Swanson et al., 2021) and a phase III study (van Dyck et al., 2023). The positive outcome of the phase III trial led the U.S. Food and Drug Administration to give Lecanemab full approval for the treatment of AD in July 2023.

Preclinical studies showed that intravenous injection of a tau oligomer-specific monoclonal antibodies in tau P301L transgenic mice reversed motor deficits, successfully removing oligomeric tau from the animal brains (Castillo-Carranza et al., 2014). The same group later published another study employing either the same or another clone of tau oligomeric antibodies in aged Htau and JNPL3 mouse models, showing that intravenous injections of such mouse monoclonal antibodies can rescue the cognitive deficits in a model-dependent manner and led to the reduction of tau oligomers, but not late p-tau pathology (Bittar et al., 2022).

APN-005 is the humanized monoclonal anti-tau IgG antibody of the mouse mAb005, raised against a conformational epitope of synaptic tau oligomers. It is currently being tested in a phase I clinical trial by the company Aprinoia. A preprint from Aprinoia scientists reported that the antibody blocks pathogenic tau in cells treated with rTg4510 mouse brain extracts. A partial rescue of synaptic and neuronal loss in rTg4510 mice was observed upon treatment with mAb005, without observing a reduction in tau aggregates (Hwan-Ching et al., 2022).

The mode of action for these therapeutic antibodies is unclear, but it could be speculated that soluble oligomers are sequestered with or without subsequent degradation in neurons and/or glial cells. Such antigen–antibody reactions could potentially take place either inside or between the cells. In either way, the decreased presence of oligomers could lead to a decreased efficiency in seeding and propagation and, thereby, reduced development of further protein pathology.

In addition to immunotherapies with monoclonal antibodies, therapeutic approaches employing small molecules to target protein misfolding and aggregation have been proposed (Gadad et al., 2011; Gabr and Peccati, 2020; Lo Cascio et al., 2020), and some compounds are currently being tested in clinical trials. The inhibitory activity of some of these drug candidates has been associated with them targeting oligomers or oligomerization. For example, prevention of α-syn oligomerization or aggregation in synucleinopathies is the target of the compounds minzasolmin (UCB0599; Price et al., 2018, 2023) and emrusolmin (Anle138b; Fellner et al., 2016; Heras-Garvin et al., 2019), currently in or planned for phase II trials for the treatment of PD or MSA, respectively. ALZ-801, a prodrug of homotaurine, its metabolite 3-sulfopranpanoic acid (3-SPA) and PRI-002 were also found to inhibit the aggregation of Aβ42 into oligomers (Gervais et al., 2007; Kocis et al., 2017; Hey et al., 2018). The prodrug is currently in a phase III trial enrolling participants with early to mild AD homozygous for ApoE4 (Alzheon, 2021; press release), while a phase II trial for PRI-002 was planned to start at the beginning of 2024. Several compounds have been tested to prevent tau aggregation for the treatment of AD and other tauopathies. OLX-07010 and TRx0237 (LMTX^™^) were reported to prevent tau self-association and inhibit the formation of oligomers (Tian et al., 2013; Harrington et al., 2015), showing promising efficacy in preclinical models (Melis et al., 2015; Davidowitz et al., 2023). Currently, OLX-07010 is being tested in a phase I trial, while TauRx is now pursuing marketing authorization for LMTX™ in the United Kingdom.

Even though it is incompletely understood by which mechanisms that various compounds can target oligomers therapeutically it is conceivable that they may, at least partly, do so by interfering with their seeding and propagation effects.

## 9 Discussion/conclusion

Similar to the prion protein, other proteins involved in neurodegeneration such as α-syn, Aβ, and tau can adopt toxic conformations that can seed and propagate pathology within the brain. While most studies have investigated the seeding properties of larger assemblies, such as fibrils, a growing number of studies have identified oligomeric assemblies to also contribute to seeding and propagation of protein aggregates. In particular, the intermediately sized oligomers/protofibrils have not only been proven to exhibit cellular toxicity, but also to be seeding and propagation competent.

Studies on oligomers are challenging because of the instability and transient nature of such species. For example, the type and characteristics of oligomers are likely to be influenced by the isolation methods. Although the conditions that enhance the seeding and propagation of oligomers are likely to vary between different amyloidogenic proteins, the majority of studies have suggested that larger oligomers (generally >400 kDa) are more seeding and propagation prone than those that are smaller in size. It must be kept in mind, however, that there might not always be a correlation between toxicity and the seeding capacity of a certain species.

Besides the intrinsic characteristics of oligomeric assemblies, differences in experimental designs to assess their seeding properties can be an additional source of variability. Some studies have reported different outcomes when the same species have been studied in different experimental settings. As suggested by the authors of one such study, the stability of the preparations and the abundance of available templates might explain the differences observed *in vitro* and *in vivo* (Fritschi et al., 2014). However, more research is needed until we can more fully understand the factors governing these processes.

Another understudied aspect is the potential for cross-seeding between different amyloidogenic proteins. Indeed, co-pathologies are observed in the majority of neurodegenerative diseases but the extent and nature of cross-talk between different pathogenic proteins is not fully elucidated. While we have here described several studies addressing co-seeding and co-aggregation *in vitro* and using preclinical models, the extent by which such events occur in the human brain is uncertain. More research aimed at understanding these mechanisms would not only increase our knowledge of underlying disease mechanisms, but also have diagnostic and therapeutic implications. For example, a treatment strategy directed toward aggregates of a specific protein might not be effective on heterologous aggregates or would require to also target the other species involved. Thus, possible strategies to target such co-pathologies may require dual approaches, such as immunotherapies and inhibitors directed toward more than one target (Subedi et al., 2022).

Taken together, continued research on the prion-like behaviors of oligomeric forms of amyloidogenic proteins would not only expand our knowledge of underlying disease processes but also offer us novel diagnostic and therapeutic possibilities for neurodegenerative disorders.

## Author contributions

SZ: Visualization, Writing – original draft, Writing – review & editing. SG: Writing – original draft, Writing – review & editing. GG: Writing – review & editing. JW: Conceptualization, Writing – original draft, Writing – review & editing. MI: Conceptualization, Writing – original draft, Writing – review & editing.
